# Prevention of Overweight and Obesity: How Effective is the Current Public Health Approach

**DOI:** 10.3390/ijerph7030765

**Published:** 2010-02-26

**Authors:** Ruth S.M Chan, Jean Woo

**Affiliations:** Department of Medicine & Therapeutics, Chinese University of Hong Kong, Hong Kong, China; E-Mail: jeanwoowong@cuhk.edu.hk

**Keywords:** obesity, prevention, public health

## Abstract

Obesity is a public health problem that has become epidemic worldwide. Substantial literature has emerged to show that overweight and obesity are major causes of co-morbidities, including type II diabetes, cardiovascular diseases, various cancers and other health problems, which can lead to further morbidity and mortality. The related health care costs are also substantial. Therefore, a public health approach to develop population-based strategies for the prevention of excess weight gain is of great importance. However, public health intervention programs have had limited success in tackling the rising prevalence of obesity. This paper reviews the definition of overweight and obesity and the variations with age and ethnicity; health consequences and factors contributing to the development of obesity; and critically reviews the effectiveness of current public health strategies for risk factor reduction and obesity prevention.

## 1. Introduction

Obesity is a public health problem that has raised concern worldwide. According to the World Health Organization (WHO), there will be about 2.3 billion overweight people aged 15 years and above, and over 700 million obese people worldwide in 2015 [1]. Although a few developed countries such as the United Kingdom and Germany experienced a drop in the prevalence rate of obesity in the past decade, the prevalence of obesity continues to rise in many parts of the world, especially in the Asia Pacific region [2,3]. For example, the combined prevalence of overweight and obesity increased by 46% in Japan from 16.7% in 1976–1980 to 24.0% in 2000, and by 414% in China from 3.7% in 1982 to 19.0% in 2002 [4].

An exhaustive body of literature has emerged to show that overweight and obesity are major causes of co-morbidities, including type II diabetes, cardiovascular diseases, various cancers and other health problems, which can lead to further morbidity and mortality [5,6]. The related health care costs are also substantial. In the United States, the total costs associated with obesity accounted for 1.2% gross domestic product (GDP) [7]. In Europe, up to 10.4 billion Euros was spent on obesity-related healthcare, and the reported relative economic burdens ranged from 0.09% to 0.61% of national GDP [8]. In China, the total medical cost attributable to overweight and obesity was estimated at about 2.74 billion US dollars and these accounted for 3.7% of national total medical costs in 2003 [9]. The total direct costs attributable to overweight and obesity in Canada has been estimated to be 6.0 billion US dollars (of which 66% is attributable to obesity), corresponding to 4.1% of the total health expenditure for 2006. Furthermore, if related co-morbidities were included, the direct cost increased by 25% [10].

In view of the epidemic of obesity as a global public health concern, this paper aims to discuss four topic areas: (1) definition of overweight and obesity; (2) health consequences of obesity; (3) factors contributing to the development of obesity; and (4) the effectiveness of current public health strategies for risk factor reduction and obesity prevention.

## 2. Definition of Overweight and Obesity

### 2.1. In Adults

Obesity can be defined as a condition of abnormal or excess fat accumulation in adipose tissue, to the extent that health may be impaired [11]. Body Mass Index (BMI), which is calculated as [(weight in kg) / (height in m)^2^], is considered to be the most useful population-level measure of obesity, and it is a simple index to classify underweight, overweight and obesity in adults. The WHO has classified overweight and obesity in adults based on various BMI cutoffs [11]. These cutoffs are set based on co-morbidities risk associated with BMI (Table 1). However, the use of BMI does not distinguish between weight associated with muscle and weight associated with fat, and the relationship between BMI and body fat content varies according to body build and proportion [12]. In contrast, the measure of intra-abdominal or central fat accumulation to reflect changes in risk factors for cardiovascular diseases and other forms of chronic diseases is better than BMI [13,14]. Therefore, an assessment of central fat accumulation greatly assists in defining obesity.

Numerous studies have compared the appropriateness of various anthropometric indices for assessing obesity and predicting obesity-related health risks, including BMI, waist-to-hip ratio (WHR), waist circumference (WC), and waist-to-height ratio (WHtR) [14–17]. However, there is no agreement on which index should be applied universally for defining obesity.

WHR was shown to be a good predictor of health risk [18], and a high WHR (>1.0 in men and >0.85 in women) indicates abdominal fat accumulation [19]. However, the use of WHR has been recently challenged due to several reasons [14,20]. First, hip circumference could not be obtained routinely and the measure is more difficult to perform and less reliable. Second, WHR is not useful in practical risk management as the ratio could remain constant when the weight of individual increases or decreases.

A health risk classification based on WC is suggested to be more useful for health assessment than either BMI or WHR, alone or in combination [19,21–23]. Data from a random sample of 2,183 men and 2,698 women aged 20−59 years from the Netherlands indicated that a WC greater than 102 cm in men, and greater than 88 cm in women, is associated with a substantially increased risk of obesity-related metabolic complications (Table 2) [24]. The relation between WC and clinical outcome is consistently strong for diabetes risk, coronary heart diseases, and all-cause and selected cause-specific mortality rates, and WC is a stronger predictor of cardiometabolic risks than is BMI [13]. In Chinese adults, the best anthropometric measurements to screen for metabolic syndrome is WC, since it was better associated with metabolic risk factors than BMI, WHR and WHtR [14]. However, the influence of the optimal cutoff values of WC by sex, age and race-ethnicity as suggested by previous studies raises the problem of applying WC for obesity assessment (Table 3) [14,25,26].

WHtR has been proposed as another rapid and simple screening tool for assessing obesity [27]. WHtR values above 0.5 indicate increased risk and values above 0.6 indicate substantially increased risk [20]. Results of a meta-analysis showed that WHtR was better than WC, WHR, and BMI for detecting cardiovascular risk factors in both men and women [28]. The results were also supported by prospective studies [15,27]. An advantage of using WHtR over WC for assessing obesity is that the same cutoffs can be set for men and women, for children and adults, and for different ethnic groups [27].

There are ethnic variations in the association between adiposity and health, and Asian populations are generally more susceptible to the development of obesity-related illnesses and morbidity than Caucasian populations at any given level of BMI or WC [3,29–31]. A meta-analysis among different ethnic groups also showed that body fat percentage was 3−5% higher in Asian populations compared to Caucasian populations for the same BMI, and BMI was 3−4 units lower in Asian populations compared to Caucasian populations for the same body fat percentage [32]. These variations in the association between BMI or WC and risk of obesity-related illnesses and morbidity, and between BMI and body fatness have raised the need for population-specific BMI and waist classification cutoff points for defining obesity. A lower BMI cutoff points for overweight (≥23.0 kg/m^2^) and obesity (≥25.0 kg/m^2^) for Asians [11], and a series of ethnic-specific WC cutoff points to define abdominal obesity (Table 3) [25] were proposed. However, the cutoff point for observed risk varies from 22.0 to 25.0 kg/m^2^ in different Asian populations; and for high risk it varies from 26.0 to 31.0 kg/m^2^. Therefore, the WHO Expert Consultation recommended that the current WHO BMI cutoff points (Table 1) should be retained as the international classification [33].

### 2.2. In Children and Adolescents

Defining overweight and obesity in children and adolescents is complicated as height is still increasing and body composition changes over time. Different measures and references such as weight-for-height, BMI percentiles, and skinfold thickness have been used [11,34]. Recently, BMI has been increasingly accepted as a valid indirect measure of adiposity in children and adolescents [35,36]. Cole *et al.* (2000) [35] published a set of smoothed sex-specific BMI cutoff values based on six nationally representative data sets from Brazil, Great Britain, Hong Kong, the Netherlands, Singapore and the United States. The proposed BMI cutoff value for overweight was 25 kg/m^2^ and for obesity was 30 kg/m^2^ at age 18 years averaged across the six populations. However, the reference data sets do not adequately represent non-Western populations, and little is known about whether or not BMIs above these cutoff points are related to health consequences for children across populations. Therefore, from 2006 onwards, the WHO released two new sets of growth standards for infants and young children [37], and school aged children and adolescents [38], respectively. The standards for infants and young children was developed based on healthy, breast-fed children from around the world [39,40]. The reference for school aged children and adolescents was developed from reconstructing the 1977 National Center for Health Statistics/WHO growth reference from 5 to 19 years, supplemented with data from the WHO Child Growth Standards, and applying the state-of-the-art statistical method [39,41]. A recent international survey also proposed a lower cutoff BMI value of 17 as definition of thinness in children and adolescents [42].

### 2.3. In Elderly

With aging, body composition changes and height decreases, affecting the interpretation of anthropometric data. Older persons generally have more fat than younger adults do at any given BMI, and absolute levels of WC indicate more visceral fat in older persons than in younger persons, because relatively more fat accumulates in the abdomen and less fat at the extremities as people age [43]. In general, BMI is a common method to diagnose obesity in older adults, but because of height and body composition changes with ageing, the cutoff values applied to adults might have to be reconsidered in old subjects [44,45]. In contrary to the young or middle-aged population, numerous studies have reported a J- or U-shaped relationship between BMI and mortality in older adults, and underweight is hazardous whereas mild-grade overweight, obesity and even central obesity might be protective for older adults [46–48].

Due to the progressive age-decline in stature, using BMI to classify obesity may overestimate adiposity in the elderly [49]. Furthermore, BMI cannot make a discrepancy between fat and muscle mass [45]. The reliability of BMI as an index of obesity is thus questionable, and therefore, other anthropometric indices are proposed to determine the degree of fatness in the elderly. These indices include WC, WHR, WHtR and sagittal abdominal diameter. However, the choice of measurement and the cutoff values in predicting mortality or other cardiovascular risks in the elderly population is still uncertain [50–53].

In summary, since the associations between adult values for overweight and obesity and certain adverse health outcomes in elderly populations show conflicting results with a suggestion that higher values may not result in adverse health outcomes, it may not be appropriate to apply existing adult values to elderly people aged 70 year and over. In view of the rapidly growing numbers of people in this age group in many developed countries with population ageing, this has important health implications in terms of health promotion and treatment targets. Further research is indicated in establishing criteria for a healthy weight in people aged 70 years and over, using relevant health outcomes such as functional independence in addition to disease occurrence. The emphasis may likely be on weight maintenance rather than reduction at the extreme of old age, when ability to modify lifestyle may be limited and quality of life may assume greater importance.

## 3. Health Consequences of Obesity

Numerous epidemiological studies have been conducted to show the relationship between excess weight, abdominal fatness and risk of a wide range of illnesses [6,54–56]. Table 4 summarizes the approximate relative risk of physical health problems associated with obesity [57].

### 3.1. Diabetes

Of all physical health problems, type II diabetes has the strongest association with obesity. A meta-analysis examined the relative risk of incidence of various co-morbidities related to obesity and overweight from 89 studies [6]. Elevated BMI and WC were significantly associated with incidence of type II diabetes in men and women. Obesity, as defined by BMI, showed the strongest association with incidences of type II diabetes as compared to other co-morbidities. The pooled relative risks (95% confidence interval) across categories of BMI were 6.75 (5.55–8.19) in men and 12.41 (9.03–17.06) in women [6]. In the Nurses’ Health Study, which followed 78,419 apparently healthy women for 20 years, for each 5-unit increment in BMI, the multivariate relative risk (95% confidence interval) of diabetes was 2.36 (1.83–3.04) for Asians, 2.21 (1.75–2.79) for Hispanics, 1.96 (1.93–2.00) for whites, and 1.55 (1.36–1.77) for blacks [58].

### 3.2. Cardiovascular Diseases

Obesity predisposes an individual to a number of cardiovascular risks including hypertension, dyslipidemia and coronary heart disease [6,59]. In the Multi-Ethnic Study of Atherosclerosis, which assessed the association between obesity and cardiovascular risk factors and subclinical vascular disease in 6,814 persons aged 45 to 84 years, showed that a higher BMI was associated with more adverse levels of blood pressure, lipoproteins, and fasting glucose, and higher prevalence ratios of hypertension [60]. Another study in an Asia Pacific population reported that a one-standard deviation increase in index was associated with an increase in risk of ischemic heart disease of 17% (95% CI 7–27%) for BMI, 27% (95% CI 14–40%) for WC, 10% (95% CI 1–20%) for hip circumference, and 36% (95% CI 21–52%) for WHR [61].

### 3.3. Cancers

A number of reviews have considered the association of obesity and cancer [6,62–64]. Data from a meta-analysis showed that the pooled relative risks across categories of BMI for various cancers ranged from 1.05–2.29 in men and 1.13−3.22 in women [6]. The recent report by the World Cancer Research Fund and the American Institute for Cancer Research (2007) [57] also suggested that there was convincing evidence that overweight and obesity increased the risk of cancers of the esophagus, pancreas, colon and rectum, breast (postmenopausal), endometrium, and kidney. In addition, there was convincing evidence to support that abdominal fatness was a cause of colon cancer and may probably increase the risk of cancers of breast (postmenopausal) and endometrium.

### 3.4. Other Health Consequences of Obesity

There is a wealth of evidence to show that excess weight is an important risk factor in the development of other illnesses, including respiratory diseases [54], chronic kidney diseases [56], musculoskeletal disorders [65,66], gastrointestinal and hepatic disorders [67,68], lower physical functioning performance [69] and psychological problems [11].

## 4. Factors Contributing to the Development of Obesity

The etiology of obesity is multifactorial, involving complex interactions among the genetic background, hormones and different social and environmental factors, such as sedentary lifestyle and unhealthy dietary habits [11]. Table 5 lists the key factors that might promote or protect against weight gain and obesity as suggested by the WHO [70].

Nutrition transition as a result of urbanization and affluence has been considered as the major cause for the obesity epidemic [70]. Numerous literatures have documented a marked shift in the dietary pattern worldwide [70,71]. Major dietary changes include a higher energy density diet with a greater role for fat and added sugars in foods, greater saturated fat intake (mostly from animal sources), marked increases in animal food consumption, reduced intakes of complex carbohydrates and dietary fiber, and reduced fruit and vegetable intake [70–73]. These dietary changes are compounded by lifestyle changes that reflect reduced physical activity at work and during leisure time [71,74]. Several studies have shown that insufficient physical activity is one of the important risk factors of obesity [75–77], and work-related activity has declined over recent decades in industrialized countries whereas leisure time dominated by television viewing and other physically inactive pastimes has increased [71,74].

Social inequality as a result of economic insecurity and a failing economic environment is also considered as one of the probable causes of obesity. A review by Drewnowski (2009) [78] indicates that inequitable access to healthy foods as determined by socioeconomic factors could influence the diet and health of a population. Energy-dense and nutrient-poor foods become the best way to provide daily calories at an affordable cost by the poor groups, whereas nutrient-rich foods and high-quality diets not only cost more but are consumed by more affluent groups. Lack of accessibility of healthy food choices [79] and the commercial driven food market environment [80] are also considered as other probable causes of obesity.

The interaction effects among environmental factors, genetic predisposition and the individual behavior on excess weight gain has received research interests in recent decades. “Gene-environment interaction” refers to a situation in which the response or the adaptation to an environmental agent, a behavior, or a change in behavior is conditional on the genotype of the individual [81]. Observational evidence has shown that susceptibility to obesity is determined largely by genetic factors, but the environment prompts phenotype expression. For instance, a study of 285 healthy Japanese men indicated that a missense variant in the interleukin 6 receptor gene interacted significantly with dietary energy intake levels in relation to the risk of abdominal obesity [82]. In a cross-sectional study of 632 men, it was found that intake of total fat and saturated fatty acids was significantly associated with WC in individuals with the *PRARα* Leu162/Leu162 genotype, but not in those with the Val162 allele [83]. Possible mechanisms by which genetic susceptibility may operate include low resting metabolic rate, low rate of lipid oxidation, low fat-free mass and poor appetite control [11].

An adverse environment during *in utero* or postnatal periods has also been suggested as one possible cause for the development of obesity, indicating that the mother’s nutrition or perinatal lifestyle could affect the developmental programming of the fetus. The concept of programming in fetal or postnatal life is firstly established from experimental animal studies. A wealth of evidence from animal studies has demonstrated that exposure to an elevated or excess nutrient supply before birth is associated with an increased risk of obesity and associated metabolic disorders in later life [84]. Results from epidemiological studies and experimental studies in human also supported that intrauterine or postnatal nutrition could predispose individuals to obesity in later life [84,85]. In a review by Martorell and colleagues (2001) [85], intrauterine over-nutrition as proxied by high birth weight or gestational diabetes is associated with subsequent fatness, and breastfeeding has a protective effect on the development of obesity. In contrast, the evidence that poor nutrition in early life is a risk factor for increased fatness later in life is still inconclusive.

## 5. Effectiveness of the Current Public Health Strategies for Risk Factor Reduction and Obesity Prevention

A public health approach to develop population-based strategies for the prevention of excess weight gain is of great importance and has been advocated in recent years [11,86]. The development and implementation of obesity prevention strategies should target factors contributing to obesity, should target barriers to lifestyle change at personal, environmental and socioeconomic levels, and actively involve different levels of stakeholders and other major parties. A proposed framework by Sacks (2009) [87] suggests that policy actions to the development and implementation of effective public health strategies to obesity prevention should (1) target the food environments, the physical activity environments and the broader socioeconomic environments; (2) directly influence behavior, aiming at improving eating and physical activity behaviors; and (3) support health services and clinical interventions. Examples of policies under each of these groups are reviewed in the following sections.

### 5.1. Food, Physical Activity, and Socioeconomic Environments

To alter the food environment such that healthy choices are the easier choices, and to alter the physical activity environment to facilitate higher levels of physical activities and to reduce sedentary lifestyle, are the key targets of obesity prevention policies. There are a wide range of policy areas that could influence the food environments. These areas include fiscal food policies, mandatory nutrition panels on the formulation and reformulation of manufactured foods, implementation of food and nutrition labeling, and restricting marketing and advertising bans of unhealthy foods [87–89]. For instance, some studies have demonstrated that food prices have a marked influence on food-buying behavior. A small study was done in a cafeteria setting and was designed to look at the effects of availability and price on the consumption of fruit and salad. It was shown that increasing variety and reducing price by half roughly tripled consumption of both food items, whereas returning price and availability to the original environmental conditions brought consumption back to its original levels [90]. A larger study designed to look at the effects of health education and pricing on the consumption of vending machine snacks also showed similar results, in which price reductions on low-fat items increased the proportional purchase of low-fat items by 9%, 39%, and 93% in the 10%, 25%, and 50% price reduction conditions, respectively [91].

Policy areas influencing physical activity environments include urban planning policies, transport policies and organizational policies on the provision of facilities for physical activity [87,92]. A recent review by Sallis and Glanz (2009) [93] summarized the impact of physical activity and food environments as solutions to the obesity epidemic. Living in walkable communities and having parks and other recreation facilities nearby were consistently associated with higher levels of physical activity in youth, adults, and older adults. Better school design, such as including basketball hoops and having a large school grounds, and better building design, such as signs promoting stair use and more convenient access to stairs than to elevators were associated with higher levels of physical activity in youth, adults and older adults [93].

As mentioned earlier, social inequality as a result of economic insecurity and a failing economic environment is also considered as one of the probable causes of obesity [78]. Therefore, policy areas covering the financial, education, employment and social policies could impact population health. As illustrated by Sacks (2009) [87], trade agreements between countries, personal income tax regimes and social security mechanisms are some potential policy areas that could be altered at international, national and state levels for the development of population-based strategies for obesity prevention.

### 5.2. Influencing Eating and Physical Activity Behaviors

According to Sacks’ framework (2009) [87], policies that directly influence behaviors need to have a direct effect in the settings in which people live their lives. There are many key settings, such as schools, home environment, workplaces and community, in which policies could target to directly influence the eating and physical activity behaviors.

A policy-based school intervention has been found to be effective for the prevention and control of obesity. The two-year School Nutrition Policy Initiative including components of school self-assessment, nutrition education, nutrition policy, social marketing, and parent outreach has been documented to be effective in reducing the incidence of overweight in school children [94]. A review examined the effectiveness of school-based strategies for obesity prevention and control based on results of nineteen included studies [95]. Pooled results of these studies showed that nutrition and physical activity interventions resulted in significant reductions in body weight compared with control (standardized mean difference (SMD) = −0.29, 95% confidence interval (CI) = −0.45 to −0.14). Parental or family involvement of nutrition and physical activity interventions also induced weight reduction (SMD = −0.20, 95% CI = −0.41 to 0.00). A study has evaluated the effectiveness of an intervention program, based on the Theory of Planned Behavior, on obesity indices and blood pressure in Ioannina, Greece [96]. In this study, 321 fifth grade students were assigned to the one-year school-based intervention focused on overcoming the barriers in accessing physical activity areas, increasing the availability of fruits and vegetables and increasing parental support, and 325 students served as control group. After the one-year follow up, a significantly higher consumption of fruits and lower consumption of fats/oils and sweets/beverages was observed in the intervention group compared with the control group. The intervention group also showed significantly lower BMI and blood pressure than the control group. The leadership role for schools in promoting physical activity in children and youth has also been advocated in a Scientific Statement from the American Heart Association Council [97]. The Statement points out that schools are potentially attractive settings in which to promote positive health behaviors because students spend large amounts of time in the school environment, elements of the traditional school curriculum relate directly to health, and schools typically provide extracurricular programs that can promote health.

The home environment is undoubtedly an important setting in preventing overweight and obesity. Television viewing has been identified as an independent risk factor for obesity [57]. Potential strategies to reduce television time include messages to parents about not having a television in children’s bedrooms, encouraging family rules restricting television viewing, and not having the television on during dinner [98]. Other potential areas to target in terms of the home food and physical activity environment include purchasing healthy foods, practicing regular meal times, allocating individual portions, creating opportunities for physical activities, and the parents as role models for healthy eating [99]. Other potential settings for interventions include restaurants, cafeterias and other food-service settings [100], supermarkets [101], and workplaces [102]. The constructs of interest include the availability and price of healthy food choices, quality of food, portion sizes, within-outlet promotions, and point-of-choice nutrition information [93].

### 5.3. Supporting Health Services and Clinical Interventions

A number of barriers to an effective obesity management program have been identified. At the physician practice level, a lack of time to address obesity during routine office visits, a lack of reimbursement, inadequate training and low self-efficacy in handling patients of excess weight are some barriers to an effective management [103,104]. At the patient level, stigmatization [105], a lack of financial incentive [106], difficulties in accessing weight management services [79] are identified as barriers to an effective management.

There are several potential policy areas in which the involvement of primary care in reducing overweight and obesity could be increased. These areas include increasing number of dietitians and nutritionists in hospitals and subsidization of weight-loss medication [87], providing professional and organizational support and training [104], and by offering financial incentives [106]. A systematic review was done to determine the existence and effectiveness of interventions to improve health professionals’ management of obesity or the organization of care for overweight and obese people [107]. Among the 18 studies involving 446 providers and 4,158 patients, no concrete conclusion could be drawn on how the management of obesity might be improved due to the heterogeneous nature of the studies. However, reminder systems, brief training interventions, shared care, inpatient care and dietitian-led treatments might all be worth further investigation.

### 5.4. Barriers to the Effectiveness of Reduction of Overweight and Obesity through a Policy Approach

Overweight and obesity prevention or reduction essentially involves lifestyle modification through behavioral change at the individual level. Policy alone is unlikely to achieve this, merely facilitating the process. However many factors act as barriers to change. For example the universal use of information technology in all settings, whether at home or work, greatly reduces physical activity [108–110]. Examples are the wide use of social networking websites such as Facebook, YouTube *etc.*; school work dependent on the internet and computer; computer-based work dominating most occupations; entertainment dependent on information technology. Social networking and enjoyment would be strong motivation for computer use at home, while work demands would necessitate continual use at work. For the majority of people, it would be difficult to counterbalance this reduction in physical activity with the technology revolution. The habit of snack consumption at the same time also predispose to overweight and obesity [111,112].

As society becomes increasingly competitive, the resulting stress may contribute to excessive eating as some people turn to food for comfort [113]. It was hypothesized that the elevated cortisol secretion, caused by stress, might disrupt the food intake regulation in humans and could result in a long-term increased energy intake and fat accumulation [114]. Unhealthy lifestyles associated with poverty are difficult to tackle through policy, unless there is poverty reduction [78]. Finally, the goals of the food industry are to maximize profit, and this aim does not necessarily coincide with public health efforts for obesity control. The food industry strategies to maximize profits include promoting larger portions, frequent snacking and the normalization of sweets, soft drinks, snacks and fast food as daily fare [115,116]. A parallel may be drawn with the tobacco industry and the strategies used to promote their products.

Ultimately, the key to controlling the obesity epidemic lies at the level of individuals, since they have to act on health promotion advice and efforts. A recent qualitative study explored a lifestyle modification program from the clients’ perspective, showing the importance of client centered care in achieving lifestyle modification [117]. Further research is needed from the individual’s perspective. Questions to be addressed include: whether avoidance of overweight and obesity is viewed with as much concern as the prevention of diseases such as cancer or ischemic heart disease; what are factors that enable individuals to increase their physical activity level and adopt a healthy diet so that long-term behavior change is achieved; and more in depth understanding of individual, interpersonal, organizational and community factors that affect this behavior in the context of different ethnicity and culture.

## 6. Conclusions

The health risks and health care costs associated with overweight and obesity are considerable. The etiology of obesity is multifactorial, involving complex interactions among genetic background, hormones and different social and environmental factors. A public health approach to develop population-based strategies for the prevention of excess weight gain should target factors contributing to obesity, should be multifaceted, and actively involve different levels of stakeholders and other major parties. Potential policy areas to the development and implementation of such strategies should cross from the home environment to a broader policy level of socioeconomic environments. However, there is likely to be many barriers towards strategies based on policies alone. The prevention and reduction of overweight and obesity depend ultimately on individual lifestyle changes, and further research on motivations for behavior change would be important in combating the obesity epidemic.

## Figures and Tables

**Table 1.** Classification of overweight and obesity in adults according to BMI.

Classification	BMI	Risk of co-morbidities
Underweight	<18.5	Low
Normal range	18.5−24.9	Average
Overweight	25.0−29.9	Increased
Obese class I	30.0−34.9	Moderate
Obese class II	35.0−39.9	Severe
Obese class III	≥40	Very severe

**Table 2.** Sex-specific WC and risk of metabolic complications associated with obesity in Caucasians.

Risk of metabolic complications	Waist circumference (cm)	
	Men	Women
Increased	≥94	≥80
Substantially increased	≥102	≥88

Source: WHO (2000) [11].

**Table 3.** Proposed WC for diagnosing the metabolic syndrome in selected country/ethnic groups.

Country/ethnic group	Waist circumference (cm)	
	Men	Women
Europeans	≥94	≥80
In the USA, the ATP III values (102 cm male; 88 cm female) are likely to continue to be used for clinical purposes South Asians	≥90	≥80
Based on a Chinese, Malay and Asian-Indian population Chinese	≥90	≥80
Japanese	≥85	≥90

Source: James (2005) [25].

**Table 4.** Approximate relative risk of physical health problems associated with obesity.

Relative risk >3	Relative risk 2−3	Relative risk 1−2
Type II diabetes	Coronary heart disease	Cancer
Gallbladder disease	Hypertension	Reproductive hormone abnormalities
Dyslipidemia	Osteoarthritis	Polycystic ovary syndrome
Insulin resistance	Hyperuricemia and gout	Impaired fertility
Breathlessness		Low back pain
Sleep apnea		Increased risk of anesthesia complications
		Fetal defects (associated with maternal obesity)

Source: World Cancer Research Fund/American Institute for Cancer Research (2007) [57].

**Table 5.** Summary of strength of evidence on factors that might promote or protect against weight gain and obesity.

Strength of evidence	Decreased risk	Increased risk
Convincing	Regular physical activity	Sedentary lifestyle
	High dietary intake of fiber	High intake of energy-dense foods
Probable	Home and school environments that support healthy food choices for children	Adverse socioeconomic conditions in developed countries
	Breastfeeding	
Possible	Low glycemic index foods	Large portion sizes
		High proportion of food prepared outside the home (developed countries)
		Rigid restraint/periodic disinhibition eating patterns
Insufficient	Increased eating frequency	Alcohol

Source: WHO (2003) [70].
